# A comparative study of platelet-rich fibrin plugs versus biphasic calcium phosphate in treating infrabony defects in patients with periodontitis: Insights from a randomized controlled trial

**DOI:** 10.4317/jced.62666

**Published:** 2025-05-01

**Authors:** Parkavi Arumugam, Mala Dixit Baburaj, Pradeep Kumar Yadalam, Carlos M. Ardila

**Affiliations:** 1 Department of Periodontics, Saveetha Dental College and Hospital, Saveetha Institute of Medical and Technical Sciences, Saveetha University, Chennai, Tamil Nadu, India; 2 Department of Periodontics, Nair Hospital Dental College, Maharashtra University of Health Sciences, India; 3 Basic Sciences Department. Biomedical Stomatology Research Group, Faculty of Dentistry Universidad de Antioquia, UdeA, Medellín, Colombia

## Abstract

**Background:**

Bone grafts and growth factors have been utilized to manage infrabony defects. Platelet-rich fibrin (PRF) has demonstrated significant potential due to its sustained release of growth factors and autologous nature. This study aimed to conduct a clinical and radiographic comparison of PRF plug as a sole graft versus biphasic calcium phosphate (BCP) (SybografTM - Plus) in treating infrabony defects in patients with chronic periodontitis.

**Material and Methods:**

Forty patients were recruited for this randomized controlled interventional study and assigned to Group A or B via computer randomization. After open flap debridement, the infrabony defects were grafted with a PRF plug in Group A (n=20) and BCP in Group B (n=20). Clinical parameters, including the Plaque Index (PI) and Gingival Index (GI), were measured at baseline, one month, three months, and six months post-surgery while Probing Pocket Depth (PPD) and Clinical Attachment Level (CAL) were measured at baseline, three months, and six months post-surgery. The radiographic depth of defect and bone fill were assessed at baseline, immediately, and six months post-surgery. Parametric tests (t-test for two groups, ANOVA for three or more groups) were employed for statistical analysis.

**Results:**

At six months post-surgery, the differences in terms of PI (*p*-value – 0.3886), GI (*p*-value – 0.1633), PPD (*p*-value – 0.3240), CAL (*p*-value – 0.2493), and radiographic bone fill (*p*-value – 0.8168) between both groups were not statistically significant.

**Conclusions:**

PRF plug as a sole grafting material has shown promising results for periodontal regeneration comparable to BCP in the treatment of infrabony defects in patients with chronic periodontitis.

** Key words:**Periodontitis, Infrabony defects, Platelet-rich fibrin, Biphasic calcium phosphate, Periodontal regeneration, Radiographic bone fill.

## Introduction

Periodontal disease is one of the most prevalent oral diseases affecting millions of individuals worldwide. According to the global burden of periodontal disease survey from 1990 to 2019, the prevalence of periodontitis has reached 1,087,367,744 cases, nearly double that of 1990 (1). It is a chronic inflammatory disease that affects the supporting structures of the tooth, initiated in response to microbial plaque biofilm. This condition leads to a disturbance in the host-inflammatory response, resulting in pocket formation and subsequent bone and tooth loss. The disease’s progression is influenced by various factors, including systemic, genetic, environmental, and local influences. Recent studies have demonstrated a pathobiology link between periodontal disease and systemic diseases through various pathways (2). This underscores the necessity for early diagnosis and prompt management at the community level. The ultimate goal of periodontal therapy has always been to regenerate lost tissues.

Periodontal regeneration is a complex biological process that unfolds under intricately coordinated biological and molecular signals (3). It involves reconstructing heterogeneous components at varying rates influenced by growth factors. Crucial to initiating periodontal regeneration is stimulating progenitor cells to differentiate, migrate, and proliferate at the site of the osseous defect. Conventional nonsurgical and surgical treatment modalities provide a limited scope for complete regeneration (4). Typically, with such treatments, healing occurs with the formation of long junctional epithelium, increasing the likelihood of pocket recurrence. Though significant advances have been made in surgical techniques and materials—such as Guided Tissue Regeneration (GTR), soft tissue grafts, bone grafts, root biomodification, and Laser Assisted New Attachment Procedure (LANAP)—achieving complete and predicTable regeneration remains elusive (5). The quest for the ideal regenerative biomaterial has led to exploring multiple new avenues.

Autografts are considered the gold standard regenerative material among bone graft substitutes due to their osteogenic potential (6). However, they are associated with morbidity, the requirement for a second surgical site, limited material availability, and a faster resorption rate. Allografts, including Freeze-Dried Bone Allograft (FDBA), Decalcified Freeze-Dried Bone Allograft (DFDBA), and xenografts, present challenges such as unpredicTable resorption rates, risks of infectious transfer, and limited patient acceptance. Alloplasts are synthetic bone graft materials readily available, biocompatible, cost-effective, and well-accepted by patients. Recent studies have highlighted using Biphasic Calcium Phosphates (BCP), a composite bioceramic consisting of a stable hydroxyapatite and a soluble beta-tricalcium phosphate phase (7). This composition endows the graft with additional benefits of bioactivity and controlled bone remodeling, ensuring scaffold stability during osteogenesis.

The clinical application of growth factors and biological mediators has recently gained attention in regenerative periodontics. Generations of platelet concentrates have been utilized in surgical techniques, as they have demonstrated a role in wound healing and maturation (8). Choukron’s Platelet Rich Fibrin (PRF), a second-generation platelet concentrate introduced in 2001, functions as an autologous growth factor reservoir composed of a slowly polymerized fibrin network interlaced with platelets, leukocytes, cytokines, and multiple growth factors such as platelet-derived growth factor, vascular endothelial growth factor, transforming growth factor beta, and insulin-like growth factor (9). It acts as a natural autologous scaffold with a high concentration of growth factors and cytokines. It stimulates mesenchymal stem cell migration, proliferation, and differentiation, resulting in periodontal and bone regeneration. Moreover, it releases pro-angiogenic and anti-inflammatory factors that promote angiogenesis and wound healing at the site of surgery.

Studies have proven that PRF, along with open flap debridement (OFD), has better clinical outcomes in comparison to OFD alone (10). Research has also analyzed the regenerative potential of PRF in combination with various bone grafts (11). In a recent study that assessed the efficacy of PRF in combination with autograft (PRF-ABG) in comparison with enamel matrix derivative in combination with autograft (EMD-ABG), for the treatment of periodontal infrabony defects, the results showed non-inferiority of PRF-ABG in comparison with EMD-ABG (12). There is scientific consensus that PRF can be used as an effective adjunct to bone grafts in the treatment of periodontal infrabony defects. However, few studies have compared the inherent regenerative potential of PRF as a sole graft with bone grafts. To the best of our knowledge, no study has investigated the regenerative potential of PRF as a sole graft in comparison with BCP for treating infrabony defects in chronic periodontitis patients. The objective of the present study was to compare clinically and radiographically the efficacy of PRF plug versus BCP as grafts in treating infrabony defects in chronic periodontitis patients.

## Material and Methods

This randomized controlled prospective parallel-group interventional clinical trial was conducted at the Outpatient Department of Periodontics at Nair Hospital Dental College, India. The trial was registered with the Clinical Trial Registry of India (CTRI/2018/01/011301) and approval from the Institutional Ethical Committee was obtained (NHDC IEC REF NO – DC/5359/Educated 22/09/2016) before the commencement of the study. The study was carried out by the ethical standards of the committee on human experimentation and the Helsinki Declaration. Only patients willing to provide audio-visual and written consent for participation in the study were included. Systemically healthy individuals aged 20 to 60 years who were diagnosed with Stage III Grade A periodontitis (13), had vital asymptomatic teeth, and presented with infrabony defects with pocket depths of ≥ 5 mm and radiographic evidence of angular bone loss were included in the study. Patients suffering from systemic diseases, those allergic to doxycycline and/or chlorhexidine, patients taking immunosuppressive drugs or drugs causing gingival enlargement, those with one-wall osseous defects, patients with poor oral hygiene maintenance or those who smoke or chew tobacco, pregnant or lactating mothers, patients with endodontically treated teeth, and patients with a history of periodontal therapy within the last 6 months were excluded. No changes were made to the trial protocol or treatment outcomes since the commencement.

After completing phase I therapy, 40 patients were allocated using a computer-generated simple randomization technique into Group A (n=20) or Group B (n=20). The principal investigator performed randomization allocation, enrollment of participants, and intervention assignment. No blinding of the randomization sequence or intervention allocation was implemented. Patients in Group A were treated with OFD followed by grafting with PRF plug as the sole graft, while patients in Group B were treated with OFD followed by grafting with BCP (Sybograf™ – Plus). The enrollment of study participants and intervention assignment after a single investigator performed randomization, and no blinding was applied in the study. Sample size calculation was determined using estimates of mean and standard deviation values from the literature (12), employing the formula {n = 2 (Zα + Zβ)² (s)² / d²} where Zα represents the variate of alpha error (a constant with a value of 1.96), Zβ is the z variate of beta error (a constant with a value of 0.84), with approximate estimates of 80% power, 5% Type I error, and 20% Type II error.

The selected participants first underwent Phase I therapy, which involved scaling and root planing. If occlusal trauma was identified, it was treated appropriately. Patients were then provided with oral hygiene instructions and were scheduled for a follow-up visit one month later. Only those who adhered to the oral hygiene instructions were selected to proceed to the surgical phase, with baseline clinical and radiographic assessments conducted. The patients were grouped to ensure that the periodontal defects were comparable between the two groups. The clinical parameters measured included the Plaque Index (PI) (14) and Gingival Index (GI) (15), which were recorded at baseline and at 1, 3, and 6 months after surgery. Additionally, Probing Pocket Depth (PPD) and Clinical Attachment Level (CAL) were assessed using the University of North Carolina-15 (UNC-15) probe at baseline, 3 months, and 6 months post-surgery. When measuring pocket depth, an acrylic stent was prepared to ensure consistent probe placement before and after surgery.

Intraoral periapical (IOPA) radiographs of the defect sites were taken using the long cone paralleling technique at baseline, immediately after surgery, and at the 6-month follow-up. The Cemento-Enamel Junction (CEJ) served as the reference point, and the Radiographic Depth of Defect (RDD) was measured from the CEJ to the base of the defect. Radiographic bone fill was assessed by comparing the baseline RDD with the RDD at the 6-month follow-up. The radiographic measurements were evaluated using digital software (Adobe Photoshop).

Surgical Procedure: The procedure began with perioral preparation using 10% povidone-iodine for scrubbing, followed by a mouth rinse with 0.2% chlorhexidine. All aseptic measures, including continuous suction, were employed to maintain a clean surgical site. Local anesthesia was administered using 2% Lignocaine Hydrochloride and 1:200,000 Epinephrine. A full-thickness mucoperiosteal flap was reflected after making a crevicular incision with a Bard-Parker No. 15 blade and reflecting the flap with a periosteal elevator. Following flap reflection, the infected tissue from the osseous defect was surgically degranulated using Gracey curettes, and thorough root planing was conducted. The site was irrigated with normal saline.

Platelet-Rich Fibrin (PRF) Plug: For Group A participants, 10 mL of venous blood was collected and centrifuged for 10 minutes at 3,000 rpm. After centrifugation, the PRF layer was obtained, and the plasma was discarded. The PRF was compressed into a plug using a sterile syringe. Pre-suturing of the defect site was performed with 3-0 black-braided silk sutures, which were left untied.

Bone Graft Material: For bone grafting, Biphasic Calcium Phosphate (SybografTM-Plus), a synthetic nanocrystalline hydroxyapatite (HA) and beta-tricalcium phosphate (β-TCP) composite graft, was used for Group B. The material, with a particle size of 600-700 microns, was gamma-sterilized and commercially obtained from Eucare Pharmaceuticals. The PRF plug was placed into the infrabony defect for Group A, while for Group B, the Biphasic Calcium Phosphate graft was utilized.

The surgical site was closed with simple interrupted sutures, and an intraoral periapical radiograph was taken immediately after the surgery. A non-eugenol periodontal dressing was applied for one week after the surgery to protect the surgical area and ensure patient comfort.

Post-Surgical Protocol: Following surgery, patients were prescribed systemic doxycycline, ibuprofen, and paracetamol. They were also instructed to use 0.2% chlorhexidine mouthwash twice daily for seven days. Sutures were removed one week after surgery, and oral hygiene instructions were reiterated. Patients were followed up at one, three-, and six-months post-surgery.

Statistical Analysis: Data were analyzed using an unpaired t-test to compare the effects of the two treatments on clinical and radiographic parameters. Repeated measures of one-way ANOVA were utilized to assess the effect of time on each treatment, followed by Tukey’s multiple comparison test to compare parameters across time points. Data are available from the corresponding author upon request.

## Results

The study involved 40 participants, with 20 assigned to Group A (OFD + PRF plug) and 20 to Group B (OFD + BCP). The trial concluded after the follow-up period, as the primary aims and objectives were achieved. All participants completed the study with no attrition loss. Among the 40 participants, 24 were male and 16 were female, with a mean age of 35 (ranging from 23 to 55). All recruited patients were compliant throughout the study. Of the forty defects, thirty (75%) were associated with posterior teeth, whereas ten (25%) were associated with anterior teeth. Of all the defects, twenty-four were located on the mesial side (60%), and sixteen were on the distal side (40%). Twenty-one defects were in the left quadrants (52%) and nineteen in the right quadrants (48%). Postoperative healing was uneventful for both groups, with no unintended events reported.

**Figure 1:**
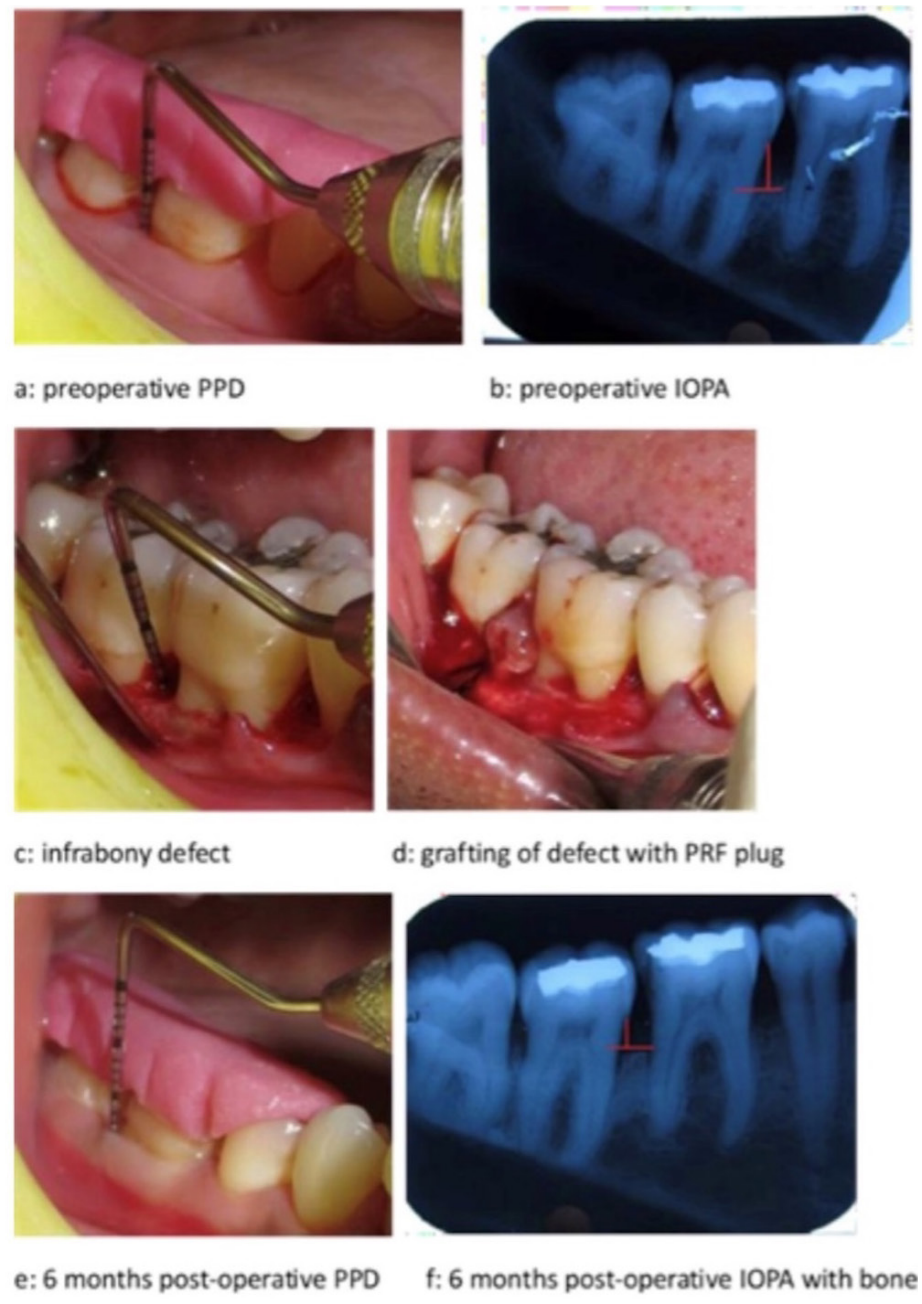
Treatment of infrabony defects with PRF plug.

**Figure 2:**
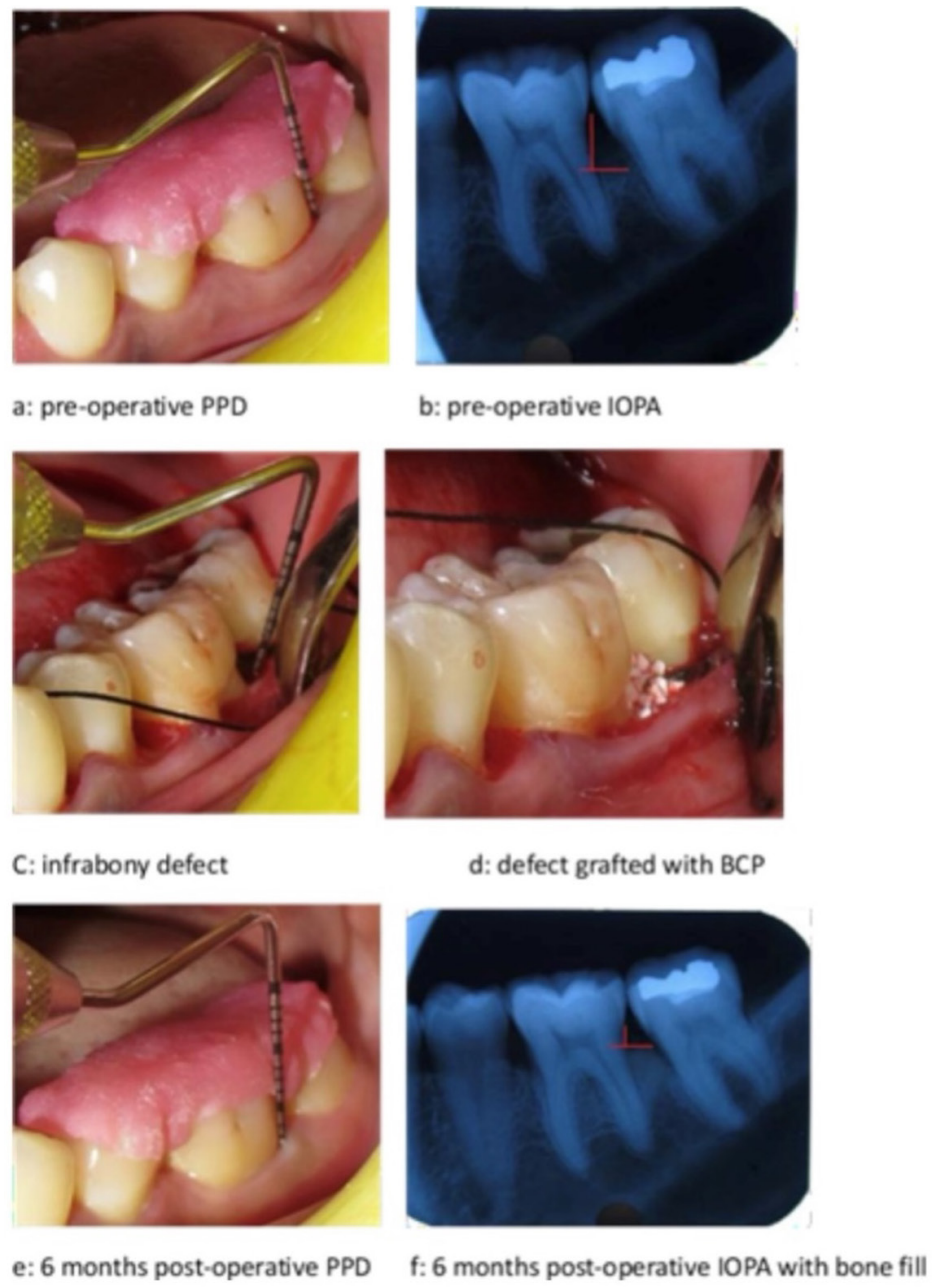
Treatment of infrabony defects with BCP.

No significant differences in baseline data were observed across all parameters between the groups. All patients adhered to the study requirements, and postoperative healing was consistent across both groups (Figs. 1,2). The overall results, including baseline recordings and outcomes, are presented in  Table 1,  Table 2,  Table 3, and  Table 4 for all parameters. Both treatment groups demonstrated statistically significant improvements in plaque and gingival index scores compared to baseline values ( Table 1, Table 2, Table 3). No statistically significant difference was noted when comparing the two groups ( Table 2).

Both treatment modalities showed statistically significant and comparable differences in PPD and CAL values at 6 months compared to baseline and 3 months post-operatively ( Table 1, Table 2, Table 3). The difference in RDD between the two groups (A and B) was statistically insignificant when observed at baseline and 6 months. However, it was found to be statistically significant immediately post-surgery, which may be attributed to the PRF plug’s radiolucent nature and the BCP’s radiopaque nature ( Table 4). There was a decrease in RDD at 6 months post-surgery when compared to baseline in both groups. When comparing both groups, there was no statistically significant difference in the radiographic bone fill, although the defect fill was slightly greater in Group A than in Group B ( Table 4).

## Discussion

Periodontal regeneration involves multiple stereochemical cues that enable specific cell populations to multiply and differentiate, thereby reconstituting lost tissue. Conventional non-surgical and surgical methods promote healing by the formation of long junctional epithelium, which tends toward re-pocket formation (16). Various regenerative techniques and materials are available, including bone grafting, guided tissue regeneration, growth factor applications, and tissue engineering. Recently, the use of growth factor concentrates like PRF, and EMD as an adjunct to bone grafting of periodontal infrabony defects, has gained attention due to improved wound healing leading to better clinical outcomes (17). However, few studies have assessed the inherent regenerative potential of PRF as a sole graft in infrabony defects. The current study aimed to assess the regenerative potential of PRF in comparison to BCP. BCP was chosen due to its bioactive nature and its composition of HA-B-TCP, allowing for balanced resorption-formation dynamics.

In the present study, no adverse post-operative complications were noted with both GROUPS A and B, demonstrating the safety of both, the PRF plug and BCP (SybografTM - Plus) for treatment of periodontal infrabony defects. No significant differences in baseline data were observed between groups, which may be attributed to the proper inclusion and exclusion criteria that minimized differences due to patient factors. Furthermore, the surgical protocol and data recording were conducted by a single investigator, eliminating potential inter-investigator variability. Thus, any differences in treatment outcomes can be attributed to the regenerative materials used.

This study evaluated PI and GI scores at baseline, 1, 3, and 6 months post-surgery. A general trend of progressive decline in PI scores was observed, with both groups showing a statistically significant reduction in PI scores from baseline to 6 months. This reduction may be attributed to repeated reinforcement of oral hygiene habits and overall improvement in periodontal parameters following surgery. However, no significant difference was observed between the two groups, indicating comparable reductions in PI scores. A similar trend of decreasing GI scores was observed for both groups at all time points compared to baseline. No significant difference was observed between the two groups, suggesting comparable reductions in GI scores. This may be attributed to the elimination of the etiologic agent and pathologic microbial burden, followed by repeated reinforcement of oral hygiene habits, allowing tissues to heal and return to a state of health.

PPD and CAL were assessed 3 and 6 months post-operatively. For both groups, mean PPD showed a significant reduction at all time points post-operatively compared to baseline, with no statistically significant difference observed between the groups. The reduction in PPD after treatment may be attributed to reduced inflammation, pocket wall shrinkage, and a combination of gain in clinical attachment and post-treatment gingival recession. Similarly, although there was a statistically significant difference in CAL at all time points compared to baseline for both groups, no significant difference in CAL gain was observed between the groups.

Of the treated defects, 75% were observed in posterior teeth, while 25% were observed in anterior teeth. This trend of increased propensity for intrabony defects (IBD) in posterior teeth can be attributed to their earlier eruption, position, complex anatomy leading to greater microbial exposure, and increased exposure to heavy masticatory forces compared to anterior teeth. Additionally, the lower accessibility of posterior teeth for daily oral hygiene maintenance may have contributed to the increased incidence of defects. Only 2- and 3-walled IBDs with a defect depth ≥ 4 mm were grafted, to achieve benefits from the added effort of grafting. The appropriate selection of IBDs would have aided in providing space maintenance and retention of the graft material within the defect during the early healing process.

The RDD was evaluated immediately and 6 months post-operatively. No significant differences in RDD were observed between groups A and B at baseline or after 6 months; however, a significant difference was noted immediately post-surgery. This difference can be attributed to the radio-opaque nature of the bone graft and the radio-lucent nature of platelet-rich fibrin. Radiographic bone fill was assessed by measuring the change in RDD from baseline to 6 months post-operatively. A significant increase in radiographic bone fill was observed at 6 months compared to baseline in both groups. However, no statistically significant difference was found when comparing the two groups. The comparable outcome observed at 6 months post-operatively may be due to the ability of PRF to induce osteoblast differentiation and bone formation, attributed to its inherent osteoinductive and osteoconductive properties. The defect fill observed in Group B may be attributed to the resorption dynamics of the bone graft material and its subsequent replacement by newly formed bone.

The results of this study are in alignment with other studies indicating good clinical outcomes with PRF (18) and BCP (19). The positive clinical outcomes noted in Group A may be attributed to the plug’s physical, mitogenic, and immune-modulatory properties. PRF serves as a reservoir of growth factors, proteins, cytokines, platelets, and leukocytes woven into a fibrin network with a tetramolecular structure. It gradually releases various growth factors sustainably, facilitating cell differentiation and migration. Additionally, it exhibits anti-inflammatory and anti-microbial properties due to the presence of leukocytes and functions as an immune regulatory node (20). Moreover, it is a fully autologous preparation with no additives, is cost-effective, and offers easy handling and molding properties, making it a preferred biomaterial for treating infrabony defects. Similarly, the positive outcomes observed in Group B may be attributed to BCP’s nanoparticle size and bioactivity enabling a synergistic bond with the defect margins, promoting new bone formation. The more sTable HA phase may have aided in preserving the augmented space, while β-TCP may have promoted bone formation. Additionally, the comparable radiographic bone fill observed in both Group A and B may be attributed to the positive regenerative ability of the PRF plug to induce osteoblast differentiation and bone formation, while the defect fill observed in Group B may be related to the resorption dynamics of the bone graft material.

Recent advancements have led to using growth factors for improved regenerative outcomes. A study compared different PRF preparation protocols using various collection tubes and compression techniques, finding no statistically significant difference (21). The vital three-dimensional organization of the fibrin network and increased elasticity are attributed to PRF’s gradual and organic polymerization. Research has indicated an initial burst of growth factor release for the first 300 minutes after PRF preparation, followed by a sustained release of cytokines and growth factors lasting 7-11 days (19). In our study, the immediate grafting after PRF plug preparation capitalized on the initial burst of growth factors, promoting healing and regeneration. The easily molded PRF plug also stayed in place, preventing graft displacement and resulting in more predicTable healing outcomes.

A recent study explaining the molecular effects of PRF on bone, stated that PRF enhances bone formation by exerting osteogenic, osteopromotive and osteoconductive mechanisms (22). PRF has been shown to enhance osteoblast differentiation transcription factor namely runt-related transcription factor 2 (RUNX2) by activating the Mitogen-activated protein kinase/Extracellular signal-regulated kinase (MAPK/ERK) signaling pathway to form mineralized nodules. It has been postulated that the osteopromotive effect of PRF is due to the up-regulated expression of osteogenic genes and proteins such as Runx2, Osterix (OSX), Osteocalcin (OCN), and ERK (23). Various molecular pathways such as the Transforming growth factor beta (TGF-β) signal pathway, Bone Morphogenetic Protein/Suppressor of Mothers against Decapentaplegic (BMP/Smad), Wingless-related integration site/β-catenin (Wnt/ β-catenin), and phosphatidylinositol 3-kinase/protein kinase B (PI3K/Akt) signal pathway, have been hypothesized to play a crucial role in the osteopromotive effect induced by PRF. Moreover, the reticular fibrin structure, filled with aggregated platelets, increases rigidity and retains various cell types, creating an optimal environment for osteoconduction. PRF prolongs its effectiveness in these processes, promoting proliferation and differentiation of osteoblasts and periodontal ligament stem cells (24). A systematic review of *in vitro* studies that assessed the effect of PRF on cells, inflammation and osteoclastogenesis stated that PRF had a positive effect on cell proliferation, migration and differentiation along with expressing an anti-inflammatory effect and suppressing the expression of osteoclast marker genes tartrate-resistant acid phosphatase (TRAP), dendritic cell-specific transmembrane protein (DCSTAMP), Nuclear Factor of Activated T-Cells (NFATc), and Osteoclast-Associated Receptor (OSCAR) (25).

Several randomized controlled clinical studies compared the regenerative potential of PRF as a sole graft to open flap debridement alone (26). They found a greater reduction in pocket depth (PD), more gain in clinical attachment level (CAL), and radiographic bone fill in infrabony defects treated with PRF as the sole graft. Similar results were observed in studies comparing the regenerative potential of PRF plugs with demineralized freeze-dried bone allograft (DFDBA) in treating infrabony defects, where in PRF as a sole graft demonstrated comparable PD, CAL gain and radiographic bone fill to that achieved with DFDBA grafting. Comparable results were noted when PRF as a sole graft was compared with Perioglass, with no significant difference in regenerative potential observed among the groups (27). PRF as a sole graft has also been effectively utilized in treating furcation defects, showing significant improvement in clinical and radiographic parameters six months post-operatively (28). Another radio-histological study assessed the effect of PRF plugs as a sole graft and PRF membranes in sinus lift cases with immediate implant placement, concluding that PRF plugs aided in stabilizing a substantial volume of natural bone around the implants in the sub-sinus cavity (29). When PRF was compared to platelet-rich plasma (PRP), evidence indicated better clinical outcomes with PRF, which offers advantages such as being less time-consuming and less technique-sensitive (18).

The results of the current study align with the evidence provided by recent systematic reviews and meta-analyses. Numerous systematic reviews have concluded that PRF, both alone and in combination with bone grafts, has shown clinically significant improvements in pocket reduction, CAL gain, and radiographic bone fill in the treatment of infrabony defects, mandibular class II furcation defects, and soft tissue root coverage of recession defects, while also being completely autologous and a cost-effective complement to other surgical regenerative therapies (30).

In the present study, the extent of CAL gain attributed to the regenerated attachment apparatus could not be determined. Additionally, radiographic analysis six months post-surgery revealed filling of the radiographic defect with bone-like radio-opaque tissue indistinguishable from native bone. The limitations of this study include the lack of three-dimensional imaging of the grafted site, the lack of a longer follow-up, and the lack of histological examination of the defect post-surgery to assess the quality of the newly formed bone and the type of healing achieved. Future studies with larger sample sizes, extended follow-up durations, and advanced radiological analysis of the regenerated bone using cone-beam computed tomography (CBCT) could be undertaken.

In conclusion of the current study, although no significant differences in clinical and radiographic parameters were observed between the two groups, the CAL gain and radiographic bone fill seen with PRF plugs may be attributed to their slow and sustained release of growth factors, their dense three-dimensional fibrin structure that promotes cell and growth factor enmeshment, as well as their antibacterial effects. These factors collectively support rapid neoangiogenesis and matrix biosynthesis and induce the bone formation needed for periodontal regeneration. Furthermore, the completely autologous nature of PRF, its easy and simplified chairside preparation, and its cost-effectiveness have established it as a preferred material for grafting infrabony periodontal defects. Hence, this study proves that the PRF plug inherently possesses regenerative ability, comparable to biphasic calcium phosphate bone graft, and it can be utilised as an autologous bone replacement graft in addition to OFD to augment the body’s effort to heal and replace the lost tissues. In a world burdened with periodontal disease and its associated morbidity, the availability of this straightforward and cost-effective autologous bone replacement graft facilitates the delivery of quality healthcare to all segments of society, thereby enhancing individual health and functioning.

## Conclusions

Platelet-rich fibrin plug, when used as a sole grafting material, has shown promising results for periodontal regeneration in clinical and radiographic parameters that are comparable to biphasic calcium phosphate (Sybograf™ – Plus) in treating infrabony defects in chronic periodontitis patients. However, further studies with a larger sample size and long-term follow-up should be conducted to establish the effectiveness of PRF as a sole regenerative material.

## Figures and Tables

**Table 1:** Effect of PRF Plug (Group A) and SybografTM – Plus (Group B) on clinical parameters.

Time	Baseline	1 month post-op	3 months post-op	6 months post-op
GROUP A				
PI	1.42 ± 0.17	0.96±0.12	0.81±0.09	0.68±0.13
GI	1.43±0.17	1.00±0.10	0.82±0.07	0.77±0.08
PPD	7.35±1.39	NA	4.20±1.20	2.50±1.05
CAL	8.10±1.83	NA	4.95±1.36	3.30±1.03
GROUP B				
PI	1.40±0.18	0.97±0.10	0.81±0.05	0.71±0.06
GI	1.40±0.16	0.99±0.10	0.82±0.05	0.74±0.05
PPD	7.40±1.43	NA	4.05±1.05	2.20±0.83
CAL	8.40±1.96	NA	5.05±1.28	3.70±1.13

**Table 2:** Effect on clinical parameters - Comparison of PRF Plug (Group A) and SybografTM – Plus (Group B) - Unpaired t-test.

	P VALUE			
Time	baseline	1-month post-op	3 months post-op	6 Months post-op
PI	0.7285	0.8259	0.8070	0.3886
GI	0.6157	0.6006	0.8235	0.1633
PPD	0.9112	Na	0.6759	0.3240
CAL	0.6197	Na	0.8115	0.2493

**Table 3:** Effect of time on clinical parameters – Repeated measure ANOVA.

	P VALUE	
REPEATED MEASURES ANOVA	GROUP A	GROUP B
PI	<0.0001	<0.0001
GI	<0.0001	<0.0001
PPD	<0.0001	<0.0001
CAL	<0.0001	<0.0001

**Table 4:** Radiographic depth of a defect and radiographic bone fill.

Data summary - Radiographic Depth of the Defect				
Time		Baseline	Immediate post- op	6 months post-op
mean±SD	GROUP A	7.24±1.49	7.19±1.48	3.40±0.75
	GROUP B	7.78±1.15	3.16±1.07	3.99±1.04
Effect of treatments on Radiographic Depth of Defect (comparison of two groups) - Unpaired t-test				
		Baseline	Immediate post-op	6 months post-op
P value		0.2043	0.0000	0.0487
Effect of Time on Radiographic Depth of Defect				
Repeated measures ANOVA			GROUP A	GROUP B
P value			< 0.0001	< 0.0001
Radiographic Bone fill 6 months post-operatively			GROUP A	GROUP B
Mean ±SD			3.84±0.77	3.78±0.72
Effect on Radiographic Bone Fill - Comparison of PRF Plug (Group A) and Sybograf^TM^ - Plus (Group B) - Unpaired t-test			P value	0.8168

## Data Availability

The datasets used and/or analyzed during the current study are available from the corresponding author.
